# The reporting of pulmonary nodule results by letter in a lung cancer screening setting

**DOI:** 10.1016/j.lungcan.2022.04.009

**Published:** 2022-06

**Authors:** Jennifer L Dickson, Amyn Bhamani, Samantha L Quaife, Carolyn Horst, Sophie Tisi, Helen Hall, Priyam Verghese, Andrew Creamer, Ruth Prendecki, John McCabe, Kylie Gyertson, Vicky Bowyer, Ethaar El-Emir, Alice Cotton, Simranjit Mehta, Fanta Bojang, Claire Levermore, Anne-Marie Mullin, Jonathan Teague, Laura Farrelly, Arjun Nair, Anand Devaraj, Allan Hackshaw, Sam M Janes, SUMMIT consortium

**Affiliations:** a Lungs for Living Research Centre, UCL Respiratory, University College London, London, UK; b Centre for Prevention, Detection and Diagnosis, Wolfson Institute of Population Health, Barts and The London School of Medicine and Dentistry, Queen Mary University of London, London, UK; c University College London Hospitals NHS Foundation Trust, London, UK; d Cancer Research UK and UCL Cancer Trials Centre, University College London, London, UK; e Royal Brompton and Harefield NHS Trust, London, UK; f National Heart and Lung Institute, Imperial College London, London, UK

**Keywords:** Early detection of cancer, Solitary pulmonary nodule, Communication, Patient satisfaction

## Abstract

**Objectives:**

Pulmonary nodules are commonly found in Lung Cancer Screening (LCS), with results typically communicated by face-to-face or telephone consultation. Providing LCS on a population basis requires resource efficient and scalabe communication methods. Written communication provides one such method. Here, we assess participant satisfaction with this approach in a LCS setting and investigate characteristics associated with dissatisfaction.

**Materials and methods:**

The SUMMIT Study is a prospective observational cohort study which aims to assess the implementation of Low-Dose Computed Tomography (LDCT) scanning for LCS in a high-risk population and validate a multi-cancer early detection blood test (NCT03934866). Participants with indeterminate pulmonary nodules requiring a three-month interval LDCT were informed of their result by postal letter and given a face-to-face appointment with a study practitioner at their interval LDCT appointment. At this appointment, having previously received their results letter, participants were verbally asked questions to assess their satisfaction with, and preferences for, methods of results communication.

**Results:**

1,900 participants were included in the analysis. 82.8% (n = 1573) were satisfied with receiving their results by letter, with 2.9% (n = 55) reporting dissatisfaction. 86.3% (n = 1640) stated it was their preferred communication method and 77.3% (n = 1469) reported that their letter contained the right amount of information. Participants from less deprived socioeconomic quintiles were more likely to report that the letter contained insufficient information and individuals aged ≥ 70 years were less likely to do so.

Although 13.7% (n = 261) participants had discussed their results with their General Practitioner (GP) prior to the study visit, 83.9% (n = 219) of these participants were satisfied with receiving results by letter, with the same proportion preferring this communication method.

**Conclusion:**

We report high participant satisfaction with the reporting of pulmonary nodule results by letter in a LCS setting. We believe this provides a feasible route forward for large-scale screening programmes.

## Highlights

• High participant satisfaction with the communication of pulmonary nodule results by letter.
• Most participants would choose to receive pulmonary nodule results by letter.
• The majority felt that their results letter contained the right amount of information.
• A significant minority of participants had discussed the results with their GP.

## 1 Introduction

In UK Lung Cancer Screening (LCS) trials, 13–24% of participants are reported to have indeterminate pulmonary nodules on baseline Low-Dose Computed Tomography (LDCT) scans, requiring three-month follow-up imaging [1], [2], [3], [4]. The SUMMIT Study is a prospective observational cohort study which aims to assess the implementation of LDCT scanning for LCS in a high-risk population in North Central and East London and validate a multi-cancer early detection blood test (NCT03934866). Participants in the study with solid nodules ≥ 80 mm^3^, <300 mm^3^; ≥6mm, <8mm; larger nodules with a Brock score of < 10%, and part-solid nodules [5] underwent a three-month interval LDCT scan.

Pulmonary nodule surveillance can cause clinically significant short-term distress for a significant minority of patients [6] when experienced as a ‘near-cancer’ diagnosis. Quality of communication is therefore integral to patient-centred outcomes [7].

Here, we assess participant satisfaction with, and preferences for, a written method of communication and investigate characteristics associated with dissatisfaction.

## 2 Materials and methods

SUMMIT Study participants requiring a three-month interval LDCT were informed of their result by a postal letter containing information about the findings, need for a repeat scan in three-months’ time (by scheduled appointment), and study team contact details for further discussion by telephone if needed. Participants undergoing an interval scan had a face-to-face appointment with a study practitioner immediately prior to the scan where they had the opportunity to ask questions.

At the face-to-face appointment, participants were verbally asked how satisfied they were with receiving their results by letter, how they felt about the amount of information in the letter, and if able to choose how to receive their result, which method they would have preferred. For each question, participants were provided with a range of options for response (shown in Table 2, Table 3). Participants were also asked if they had any questions about their results letter and if they had discussed their results with their GP or a member of the study team by telephone prior to attending their face-to-face appointment.

**Table 1:** The demographic and smoking characteristics of the participants attending for a three-month interval appointment.

	Frequency (n)	Percentage (%)
Gender*		
Female	776	40.8
Male	1,124	59.2

Mean age †, (SD)		
	66.5 (6.0)	–

Age† groups		
55–59	290	15.3
60–64	446	23.5
65–69	505	26.6
70–75	454	23.9
>75	204	10.7
Missing	1	0.1

Ethnicity‡		
Asian	107	5.6
Black	66	3.5
Mixed	37	1.9
White	1,613	84.9
Other	57	3.0
Missing	20	1.1

National Index of Multiple Deprivation (IMD)*		
Quintile 1 (most deprived)	617	32.5
Quintile 2	548	28.8
Quintile 3	327	17.2
Quintile 4	301	15.8
Quintile 5 (least deprived)	96	5.1
Missing	11	0.6

Smoking status‡		
Current smoker	939	49.4
Former smoker	961	50.6

*From primary care record, †Age at time of appointment, ‡From baseline (Y0) LHC.

**Table 2:** Participant reported satisfaction of pulmonary nodule results being reported by letter.

How satisfied or dissatisfied were you with receiving your results by letter?	Frequency (n)	Percentage (%)
Satisfied	1,573	82.8
Neither satisfied nor dissatisfied	197	10.4
Dissatisfied	55	2.9
Did not receive results letter	38	2.0
Can’t remember	37	1.9

**Table 3:** Participant reported perception of how much information was included in the pulmonary nodule results letter and preferred method of contact for pulmonary nodule results.

	Frequency (n)	Percentage (%)
How do you feel about the amount of information in the results letter?		
Too much information	10	0.5
Just the right amount of information	1,469	77.3
Not enough information	204	10.7
Can’t remember	142	7.5
N/A	75	3.9

If you could have chosen how to receive your results, which of the following methods would you have preferred?		
Letter from doctor (method used)	1,640	86.3
Telephone call from a doctor	103	5.4
Telephone call from a nurse	63	3.3
Appointment with my GP	57	3.0
Appointment with a hospital doctor	37	1.9

Participants with incidental pulmonary nodules detected at baseline LDCT scan who attended for a three-month interval Lung Health Check (LHC) appointment and LDCT between 18th July 2019 and 25th June 2021 were included.

The primary outcome measure was the proportion of individuals satisfied with pulmonary nodule results communication by letter. Secondary outcome measures included participant perception of the amount of information included in the letter, their preferred method of results communication, the type of questions asked during their appointment, and the proportion who contacted the study team or their General Practitioner (GP) to discuss the results further.

Descriptive frequencies were calculated for all outcome measures with logistic regression analyses used to explore demographic and smoking characteristics associated with responses.

## 3 Results

Data were analysed for the first 1,900 SUMMIT Study participants who attended for a three-month interval LHC. 59.2% (n = 1,124) were male, with a mean age of 66.5 years (SD 6.0). Most (84.9%, n = 1,613) were of white ethnicity, nearly two thirds were from the two most deprived quintiles nationally (61.3%, n = 1,165) and half (49.4%, n = 939) were current smokers (Table 1).

82.8% (n = 1,573) of participants were satisfied with receiving their results by letter with 2.9% (n = 55) reporting dissatisfaction (Table 2). Most participants (86.3%, n = 1,640) reported the method used (letter from doctor) was their preferred choice of communication, with 5.4% (n = 103) preferring a telephone call from a doctor and 3.3% (n = 63) a nurse.

The majority (77.3%, n = 1,469) felt the letter included the right amount of information (Table 3). Participants from less deprived socioeconomic quintiles were significantly more likely to report that the letter contained insufficient information (IMD 3: aOR:1.94; 95% CI:1.26–3.00 and IMD 4: aOR:1.71; 95% CI:1.09–2.69) and those aged 70 years and above were less likely to do so (age 70–75: aOR:0.49; 95% CI:0.30–0.79 and age > 75: aOR:0.35; 95% CI:0.18–0.69). No statistical associations were identified across gender, ethnicity and smoking status.

During the LHC appointment 43.3% (n = 823) asked further questions regarding their results letter. The most common questions sought further information on pulmonary nodules (88.6%, n = 729), with remaining questions on risk of malignant transformation (29.8%, n = 245), the follow-up process (26.6%, n = 219) and radiation risk from further imaging (4.0%, n = 33).

Few participants took the opportunity to discuss their results by telephone with the study team (5.9%, n = 112) and GP (13.7%, n = 261) prior to the LHC. Of those who discussed with their GP, 83.9% (n = 219) were satisfied with receiving results by letter, with the same proportion preferring this method of communication. While not statistically significant, there was a trend for females (21.3%) to more frequently request discussion with the study team or GP, compared to males (18.5%). Older participants and those from less deprived socioeconomic quintiles were more likely to have discussed their results with their GP.

## 4 Discussion

There was high satisfaction with the communication of pulmonary nodule results by letter and the amount of information the letter provided. <3% of participants reported dissatisfaction, with the majority (86.3%) reporting they would have chosen this method over a telephone call or appointment.

Notably, all participants with pulmonary nodules were given a face-to-face appointment immediately before their interval scan, providing the opportunity to ask questions. A significant proportion (43.3%) did so, underscoring the importance of the opportunity for discussion or providing information about commonly asked questions in advance.

A significant minority (13.7%) discussed the results with their GP prior to their interval LHC appointment. Females, older participants and those from lower levels of socioeconomic deprivation were more likely to do so. Data were not available on the proportion who sought a GP consultation primarily to discuss these results, as opposed to opportunistically discussing during an unrelated consultation. Further assessment could examine this more closely and identify ways to reduce this proportion alongside considering how behaviours may differ outside of a trial setting. However, in absolute terms, the number of participants per practice who discuss results with their GP is expected to be small. Furthermore, the majority (83.9%) of those that did so were ultimately satisfied with receiving results by letter and reported that this was their preferred method.

Qualitative data from LCS in the United States suggests participants can be left dissatisfied by results communication by letter [8]. However, our results provide real-world reassurance of the acceptability of this form of communication in a UK population. Future endeavours to understand the reasons for differences in rates of satisfaction across geographical and healthcare system boundaries should be welcomed to improve LCS communication.

In conclusion, we demonstrate high participant satisfaction with the communication of a pulmonary nodule diagnosis during LCS by postal letter, providing a feasible route forward for large-scale screening programmes in the future.

## 5 Informed consent

Informed consent was obtained from all participants in the SUMMIT Study, including those participants who were included in this analysis.

## 6 Contributions

The concept of asking questions regarding satisfaction with the process of reporting results was developed by JLD and SMJ, supported by the management team for the SUMMIT Study. JLD completed the data analysis. JLD, AB and SLQ prepared the manuscript for review. All authors contributed to the development of the manuscript and approved the final version.

### CRediT authorship contribution statement

**Jennifer.L. Dickson:** Conceptualization, Methodology, Formal analysis, Investigation, Data curation, Writing – original draft, Writing – review & editing, Visualization. **Amyn Bhamani:** Writing – original draft, Writing – review & editing, Visualization. **Samantha.L. Quaife:** Methodology, Writing – original draft, Writing – review & editing, Visualization. **Carolyn Horst:** Writing – review & editing. **Sophie Tisi:** Writing – review & editing. **Helen Halll:** Writing – review & editing. **Priyam Verghesel:** Writing – review & editing. **Andrew Creamerl:** Writing – review & editing. **Ruth Prendeckil:** Writing – review & editing. **John McCabe:** Data curation, Writing – review & editing. **Kylie Gyertson:** Writing – review & editing. **Vicky Bowyer:** Writing – review & editing. **Ethaar El-Emir:** Writing – review & editing. **Alice Cotton:** Writing – review & editing. **Simranjit Mehta:** Writing – review & editing. **Fanta Bojang:** Writing – review & editing. **Claire Levermore:** Writing – review & editing. **Anne-Marie Mullin:** Writing – review & editing. **Jonathan Teague:** Writing – review & editing. **Laura Farrelly:** Writing – review & editing. **Arjun Nair:** Writing – review & editing. **Anand Devaraj:** Writing – review & editing. **Allan Hackshaw:** Writing – review & editing. **Sam. M. Janes:** Conceptualization, Writing – review & editing, Supervision.

## Declaration of Competing Interest

The authors declare the following financial interests/personal relationships which may be considered as potential competing interests: SUMMIT is sponsored and conducted by University College London and funded by GRAIL through a research grant awarded to SMJ as principal investigator. SLQ collaborates on the SUMMIT study and has received honorarium from Elsevier for writing a book chapter. AN is a member of the advisory board for Aidence BV and Faculty Science Ltd, has received a consultation fee from MSD and honorarium for travel to a conference from Takeda. AN is an Executive Committee member for the British Society of Thoracic Imaging, Lung Taskforce member for the British Lung Foundation and clinical lead for the NHS England Targeted Lung Health Checks Programme. AH has received an honorarium for an advisory bord meeting for GRAIL, a consultation fee for Evidera Inc for a GRAIL initiated project, and previously owned shares in Illumina. SMJ has received honoraria for travel, consultancy or speaking from Astra Zeneca, BARD1 Bioscience, Optellum, Jansen, Takeda, Evidera and Achilles Therapeutics.  SMJ received grant funding from Owlstone for a separate research study and has a family member who is an employee of Astra Zeneca.  AC (2) received a HEE NIHR Pre-Doctoral Clinical Academic Fellowship. All authors perceive that these disclosures pose no academic conflict for this study and declare no other relationships or activities that could appear to have influenced the submitted work.

## Appendix 1

### SUMMIT consortium members

Sam M Janes^1^, Jennifer L Dickson^1^, Carolyn Horst^1^, Sophie Tisi^1^, Helen Hall^1^, Priyam Verghese^1^, Andrew Creamer^1^, Thomas Callender^1^, Ruth Prendecki^1^, Amyn Bhamani^1^, Mamta Ruparel^1^, Allan Hackshaw^2^, Laura Farrelly^2^, Jon Teague^2^, Anne-Marie Mullin^2^, Kitty Chan^2^, Rachael Sarpong^2^, Malavika Suresh^2^, Samantha L Quaife^3^, Arjun Nair^4^, Anand Devaraj^5^,^6^, Kylie Gyertson^4^, Vicky Bowyer^4^, Ethaar El-Emir ^4^, Judy Airebamen^4^, Alice Cotton^4^, Kaylene Phua^4^, Elodie Murali^4^, Simranjit Mehta^4^, Janine Zylstra^4^, Karen Parry-Billings^4^, Columbus Ife^4^, April Neville^4^, Paul Robinson^4^, Laura Green^4^, Zahra Hanif^4^, Helen Kiconco^4^, Ricardo McEwen^4^, Dominique Arancon^4^, Nicholas Beech^4^, Derya Ovayolu^4^, Christine Hosein^4^, Sylvia Patricia Enes^4^, Qin April Neville^4^, Jane Rowlands^4^, Aashna Samson^4^, Urja Patel^4^, Fahmida Hoque^4^, Hina Pervez^4^, Sofia Nnorom^4^, Moksud Miah^4^, Julian McKee^4^, Mark Clark^4^, Jeannie Eng^4^, Fanta Bojang^4^, Claire Levermore^4^, Anant Patel^7^, Sara Lock^8^, Rajesh Banka^9^, Angshu Bhowmik^1^^0^, Ugo Ekeowa^11^, Zaheer Mangera^12^, William M Ricketts^13^, Neal Navani^4^, Terry O'Shaughnessy^13^, Charlotte Cash^7^, Magali Taylor^4^, Samanjit Hare^7^, Tunku Aziz^13^, Stephen Ellis^13^, Anthony Edey^14^, Graham Robinson^15^, Alberto Villanueva^16^, Hasti Robbie^17^, Elena Stefan^18^, Charlie Sayer^19^, Nick Screaton^2^^0^, Navinah Nundlall^4^, Lyndsey Gallagher^4^, Andrew Crossingham^4^, Thea Buchan^4^, Tanita Limani^4^, Kate Gowers^1^, Kate Davies^1^, John McCabe^1^, Joseph Jacob^1 24^, Karen Sennett^21^, Tania Anastasiadis ^22^, Andrew Perugia^23^, James Rusius^23^

### Affiliations

1. Lungs for Living Research Centre, UCL Respiratory, University College London, London

2. CRUK & UCL Cancer Trials Centre, University College London, London

3. Centre for Prevention, Detection and Diagnosis, Wolfson Institute of Population Health, Barts and The London School of Medicine and Dentistry, Queen Mary University of London, London

4. University College London Hospitals NHS Foundation Trust, London

5. Royal Brompton and Harefield NHS Foundation Trust, London

6. National Heart and Lung Institute, Imperial College, London

7. Royal Free London NHS Foundation Trust, London

8. Whittington Health NHS Trust, London

9. Barking, Havering and Redbridge University Hospitals NHS Trust, Essex

10. Homerton University Hospital Foundation Trust, London

11. The Princess Alexandra Hospital NHS Trust, Essex

12. North Middlesex University Hospital NHS Trust, London

13. Barts Health NHS Trust, London

14. North Bristol NHS Trust, Bristol

15. Royal United Hospitals Bath NHS Foundation Trust, Bath

16. Surrey and Sussex Healthcare NHS Trust, Surrey

17. King's College Hospital NHS Foundation Trust, London

18. The Princess Alexandra Hospital NHS Trust, London

19. University Hospitals Sussex NHS Foundation Trust, Sussex

20. Royal Papworth Hospital NHS Foundation Trust, Cambridge

21. Killick Street Health Centre, London

22. Tower Hamlets Clinical Commissioning Group, London

23. Noclor Research Support, London

24. Centre for Medical Image Computing (CMIC), London

We request that the collaborators are tagged for PubMed purposes
